# A conceptual view on inertial internal waves in relation to the subinertial flow on the central west Florida shelf

**DOI:** 10.1038/s41598-018-34346-2

**Published:** 2018-10-29

**Authors:** Ekaterina V. Maksimova

**Affiliations:** 1 College of Marine Science, University of South Florida, St. Petersburg, Florida 33701 USA; 2 Center for Ocean-Atmospheric Prediction Studies, Florida State University, Tallahassee, Florida 32306 USA

## Abstract

The study reported here focuses on inertial internal wave currents on the west Florida midshelf in 50 m depth. *In situ* observations showed that the seasonal shifts in stratification change both the frequency range of inertial internal waves and their modulation time scales. According to the analysis, the subinertial flow evolution time scales also undergo compatible seasonal variations, and the inertial internal wave currents appear to be temporally and spatially related to the subinertial flow. Specifically, the subinertial flow evolving on frontal-/quasi-geostrophic time scales appears to be accompanied by the near-inertial oscillations/inertia-gravity waves in corresponding small/finite Burger number regimes, respectively. The quasi-geostrophic subinertial currents on the west Florida shelf are probably associated with the synoptic wind-forced flow, whereas the frontal-geostrophic currents are related to the evolution of density fronts. Further details of this conceptual view should, however, be elucidated in the future.

## Introduction

Oceanic inertial internal waves (IIW), which include near-inertial oscillations (NIO) and inertia-gravity waves (IGW), are a subject of intense research attention because of their crucial role in energy and momentum cascades important for correct subgrid ocean and climate model parameterizations^1–3^. They are also cited as having a significant ecological role in bringing nutrients to light by means of induced mixing in the ocean^4^ and in dispersion of particles (e.g., oil-spill droplets^5^) at submesoscales. Several theoretical mechanisms have been proposed for generation and intermittency of these waves, and many modelling and laboratory studies have been conducted^6–9^. The main theoretical principle explaining IIW generation is based on the fundamental property of geophysical flows—the relaxation of any perturbation toward a state of geophysical equilibrium. The studies developed and tested numerically or in the laboratory various mechanisms for IIW emission scenarios during such adjustments, e.g., problems of initial imbalance, spontaneous emission, seeded instability, and frontogenesis. The processes were considered at many space and time scales in regimes characterized by various Rossby, Froude, Burger, and other numbers. Several coupling and mutual interaction mechanisms between the balanced flow and the IIW have also been proposed. The review articles cited provide further details and references.

**Figure 1:**
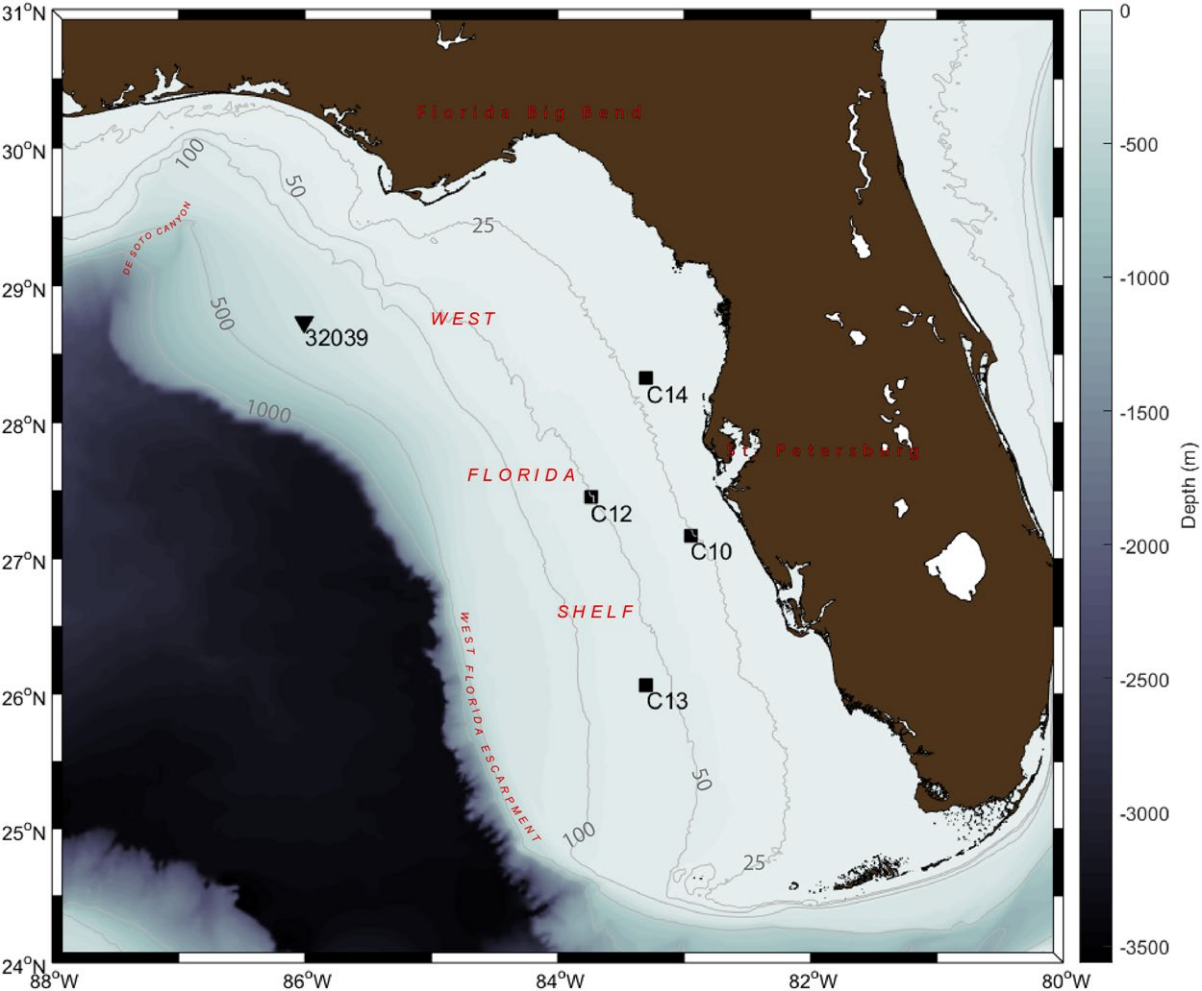
Overview of the West Florida Shelf study region in the eastern Gulf of Mexico. The *in situ* current-data stations (squares) and the wind-data station (triangle) are indicated.

Nonetheless, the past and current concepts were either not tested in real oceanic settings or the tests were only partially successful^10^ in describing the IIW currents in the ocean. Besides, except for several recent indirect demonstrations^11–13^, evidence that associates the IIW with the subinertial flow in the ocean is lacking. Our study therefore used fixed *in situ* oceanic coastal current observations to examine the relationship between the subinertial and IIW currents. We used the existing observational data on the central west Florida shelf (WFS) in the eastern Gulf of Mexico (Fig. 1). The results are presented for the data records in 50 m depth about 100 km off St. Petersburg, Florida (C12 in Fig. 1) in 2010, but similar conclusions apply at C10 (20 m), C14 (20 m), and C13 (50 m) on the central WFS as well.

## Results

### Dimensionless analysis

In theory, the oceanic IIW spectrum has a “fast” frequency range $f < {\omega }_{fast} < N$ in between the values of the Coriolis (*f* ) and Brunt-Vaisala (*N*) frequencies. The balanced state, at the next order of approximation, evolves on “slow” subinertial time scales (${\omega }_{slow} < f$), and often is regarded as a “low-frequency” current. Both subinertial and IIW current properties depend on, among other factors, the background fluid stratification (*N*), rate of fluid rotation (*f* ), and the spatiotemporal scales of the flow. If the fluid domain is characterized by some horizontal (*L*) and vertical (*H*) scales, then, by means of equations of motion scaling^14^, their relative importance is represented by the Burger number $(Bu=\frac{{N}^{2}{H}^{2}}{{f}^{2}{L}^{2}})$. The *Bu* number is a measure of stratification in the presence of rotation for a process in a given fluid domain. Considering that a fluid domain can support both subinertial and IIW currents, it is useful to distinguish and compare specific *Bu* regimes for both types of currents.

According to classic definitions^15^, the full IIW dispersion relation can be further simplified in terms *Bu* as follows. *NIO* with ${\omega }_{fast}\sim f$ correspond to the so-called hydrostatic rotating wave regime, when the Burger number is small ($Bu\ll 1$), because NIO are characterized by very small vertical to horizontal wave aspect ratios (${\alpha }^{2}=\tfrac{{H}^{2}}{{L}^{2}}\ll {\sigma }^{2}$; typically $\sigma =\tfrac{f}{N} < 1$ in the ocean). In this regime, the density stratification is effectively weak, or horizontal scale of the motion is effectively large, so rotation becomes more important:

1 $${\omega }_{fast}\sim f,\,{\omega }_{fast}^{2}={f}^{2}+{N}^{2}{\alpha }^{2}={f}^{2}(1+Bu),\,Bu\ll 1.$$

*IGW* with superinertial frequencies belong to a hydrostatic “nonrotating” wave regime for finite *Bu*. In this regime, the stratification and rotation are considered to be equally important:

2 $$f < {\omega }_{fast} < N,\,{\omega }_{fast}^{2}={N}^{2}{\alpha }^{2}=\,{f}^{2}Bu,\,1 < Bu < \frac{1}{{\sigma }^{2}}.$$

*Near*-*gravity waves* (*NGW*) with $\,{\omega }_{fast}\sim N$ must be observed at $Bu\ge \frac{1}{{\sigma }^{2}}\gg 1$ in the nonhydrostatic wave regime. Because rotational effects are not important in this regime, NGW are hereinafter ignored.

If the local subinertial *flow* is characterized by a speed scale (*U*), then the relative dynamical importances of the stratification and rotation are determined by the Froude $(Fr=\frac{U}{NH})$ and Rossby $(Ro=\frac{U}{fL})$ numbers, respectively. The subinertial flow evolves on time scales ${T}_{slow}=\frac{1}{{\omega }_{slow}}=\frac{1}{Ro\,f}=\frac{1}{Ro}{T}_{f}$, where *T*_*f*_ denotes the inertial period. Note that the three nondimensional numbers are related: $Bu={(\frac{Ro}{Fr})}^{2}$. When the flow is affected by both stratification and rotation $(Fr < 1,\,Ro < 1)$, several subinertial geostrophic flow regimes arise, according to the relative size of the isopycnal deviations. These subinertial regimes can be classified in terms of *Bu* as follows^14–16^. In a “common wisdom” *quasi*-*geostrophic* (*QG*) *regime*, the flow provides effectively small deviations of isopycnal surfaces. It is confined to domains with horizontal scales within the order of the Rossby deformation radius (finite *Bu*) and evolves on the time scales *T*_*slow*_, so:

3 $$Ro\sim Fr=\epsilon  < 1;\,Bu\sim 1;\,{T}_{slow}\sim \,\frac{1}{\epsilon }{T}_{f}.$$

When both rotation and stratification are important, the inequality $Ro\ge F{r}^{2}$ holds true, because vertical divergence cannot exist without horizontal convergence^14^. The possible parameter ranges for the QG regime at a location with $Fr=\epsilon  < 1$ therefore become:

4 $${\epsilon }^{2} < Ro < 1;\,{\epsilon }^{2} < Bu < \frac{1}{{\epsilon }^{2}};\,{T}_{f} < {T}_{slow} < \frac{1}{{\epsilon }^{2}}{T}_{f}.$$

The flow with large isopycnal deviations must have effectively large horizontal length scales (small *Bu*) and follow the so-called *frontal*-*geostrophic* (*FG*) adjustment scenario^16^. In this regime, rotational effects dominate, and the flow evolves on longer time scales:

5 $$Bu < {\epsilon }^{2};\,Ro < {\epsilon }^{2};\,{T}_{slow} > \frac{1}{{\epsilon }^{2}}{T}_{f}.$$

Note that the NIO and IGW regime parameters are consistent with those for the subinertial FG and QG regimes, respectively. Indeed, for finite *Bu* QG and IGW regimes, $O(\epsilon )\sim O(\sigma )\le O(\frac{H}{L}) < 1$. Therefore, when both subinertial and inertial motions are considered to be confined to the same fluid domain of a specific *Bu*, IGW with frequencies

6 $$f < {\omega }_{fast} < \frac{1}{\epsilon }f$$

could be intrinsic to the adjustment of the subinertial flow to the state of a new balance on QG time scales $({T}_{f} < {T}_{slow} < \frac{1}{{\epsilon }^{2}}{T}_{f})$; the *same Fr*-number subinertial flow considered on FG time scales $({T}_{slow} > \frac{1}{{\epsilon }^{2}}{T}_{f})$ could evolve together with NIO of a narrow frequency range

7 $$f < {\omega }_{fast} < \sqrt{1+{\epsilon }^{2}}\,f.$$

These ideas are tested on the basis of *in situ* oceanic coastal current and density observations in the following way.

### Observational verification

**Figure 2:**
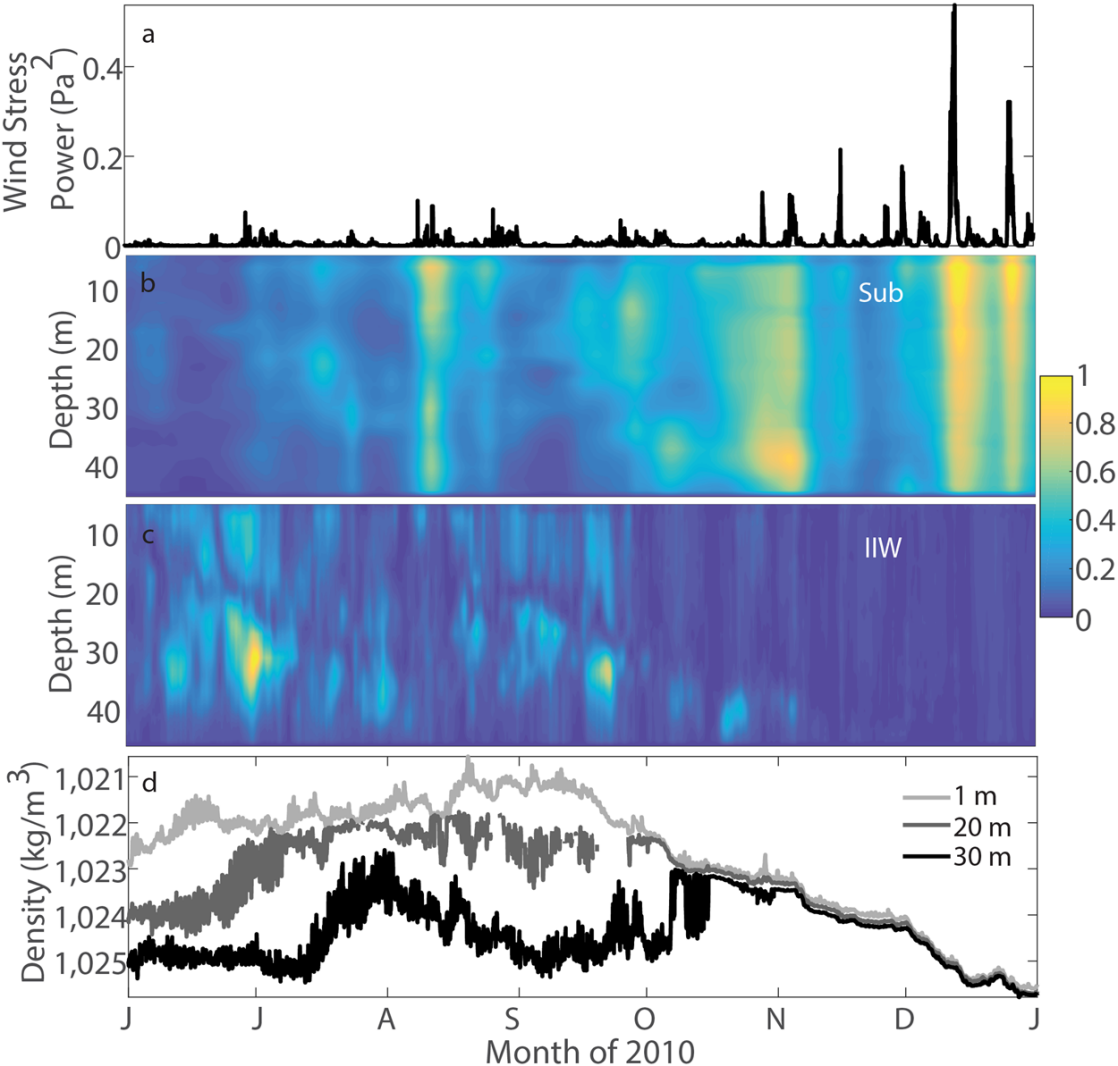
Comparison of the subinertial and IIW current power with the wind-stress power and density fluctuations at station C12 in 2010. (**a**) Wind-stress power at 42039. (**b**) Subinertial and (**c**) IIW current power at C12. The power is normalized by the maximum value for simplicity of comparison. (**d**) *In situ* density at 1, 20, and 30 m depth levels at C12.

Larger-than-usual seasonal density variations on the central WFS were measured at C12 in 2010^17^. As seen in Fig. 2d, the winter water column is nearly homogeneous ($N\sim {10}^{-3}\,{s}^{-1}$). The vertical density stratification is by an order of magnitude larger $(N \sim {10}^{-2}\,{s}^{-1})$ during summer months up until the middle of October, and that is when the IIW power (Fig. 2c) is largest as well. On the contrary, subinertial current power (Fig. 2b) stays low in summer, because subinertial flow is seen to be dominated by the wind-driven fluctuations during more frequent and energetic wintertime storms (Fig. 2a). Unlike the subinertial variability, the IIW power is poorly associated with the wind stress power input throughout the water column (Fig. 2a and c). In fact, the IIW power appears to be largest when the subinertial flow is present but is not too energetic (Fig. 2b and c). Similar behavior is observed in the laboratory during the subinertial flow adjustment; e.g., see^18^. Could evidence in Fig. 2, then, indicate that the IIW are related to processes of subinertial flow adjustment rather than the wind forcing or stratification alone?

According to (6) we can expect IGW of frequencies up to ~2*f* in winter (using winter observations, $Fr=\epsilon =\tfrac{U}{NH}\approx 0.35;\,{\rm{so}}\,f < {\omega }_{fast} < 2.8f$) and up to ~4*f* in summer (using summer observations, $Fr=\epsilon \approx 0.24;$ ${\rm{so}}\,f < {\omega }_{fast} < 4.2f$).

**Figure 3:**
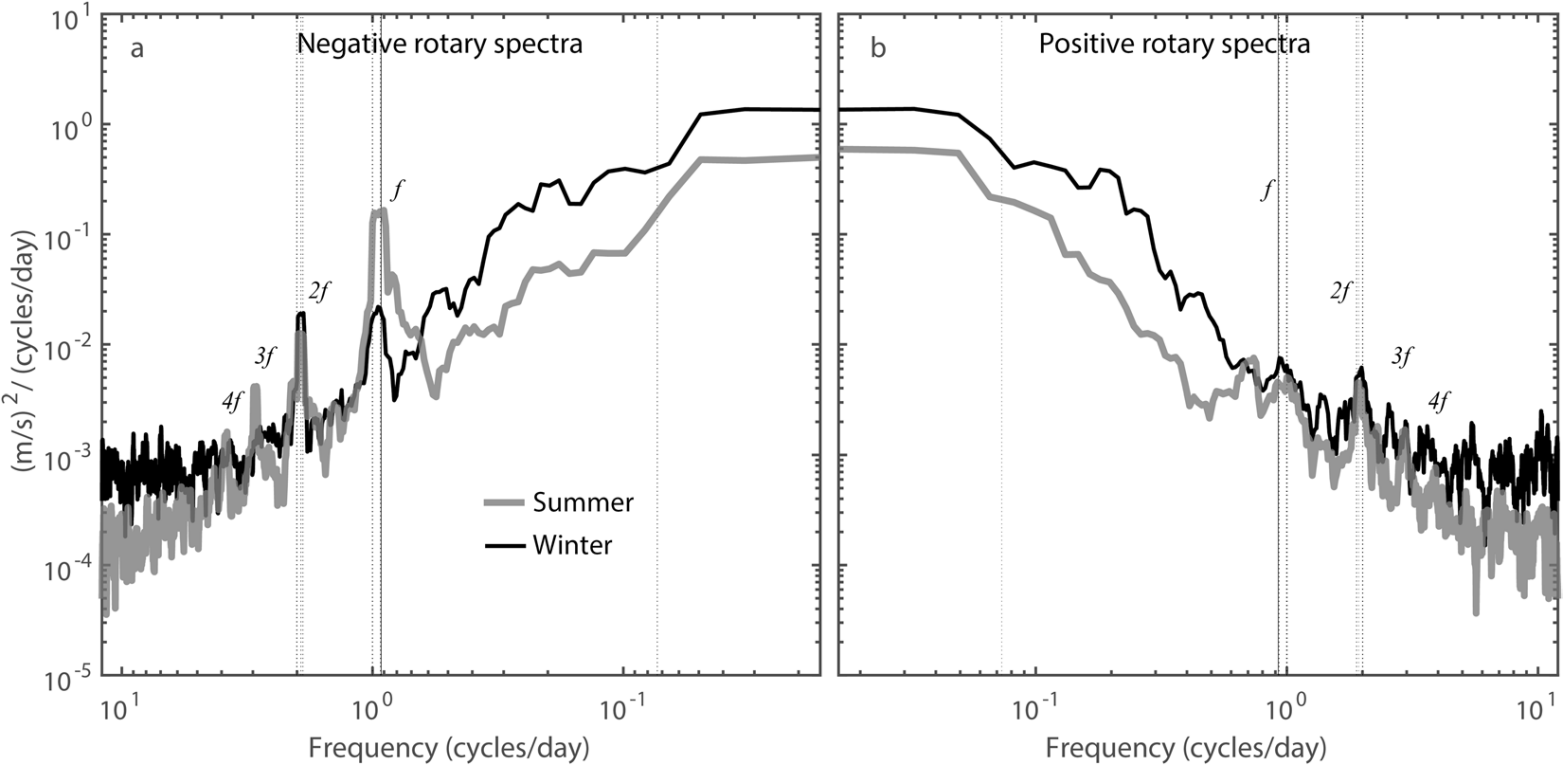
Rotary power spectral density for the current fluctuations at C12 in 2010. Depth-averaged (**a**) negative and (**b**) positive power spectral density for the current fluctuations in summer (grey) and winter (black). The inertial frequency *f* is marked by the solid vertical line at ~0.92 cycles/day. The tidal constituents are marked by dotted vertical lines and correspond to Mf, O1, P1&K1, N2, M2, S2&K2 components from right to left in the negative rotary spectra panel. The order is reversed in the positive rotary spectra panel. The indicated barotropic tidal constituents are filtered out from the current time series.

This expectation is fulfilled. Figure 3 presents the average negative rotary current spectra, where summer (grey curve) superinertial peaks are clearly present up to a ~4*f* frequency, whereas only one peak at ~2*f* is evident for winter period (black curve).

Furthermore, according to (5), NIO are expected to be associated with the low-frequency flow evolution on FG time scales of the order ~8 days in winter ($$\,Ro < 0.12;\,{T}_{slow} > 8.1\,{T}_{f}$$) and ~17 days in summer ($$Ro < 0.06;$$$${T}_{slow} > 17.3\,{T}_{f}$$). On the basis of (3) and (4), subinertial currents with shorter periods should be related to IGW and have QG time scales of the order ~2.8 and ~4.2 days in winter and summer, respectively. This expectation is also fulfilled as follows.

**Figure 4:**
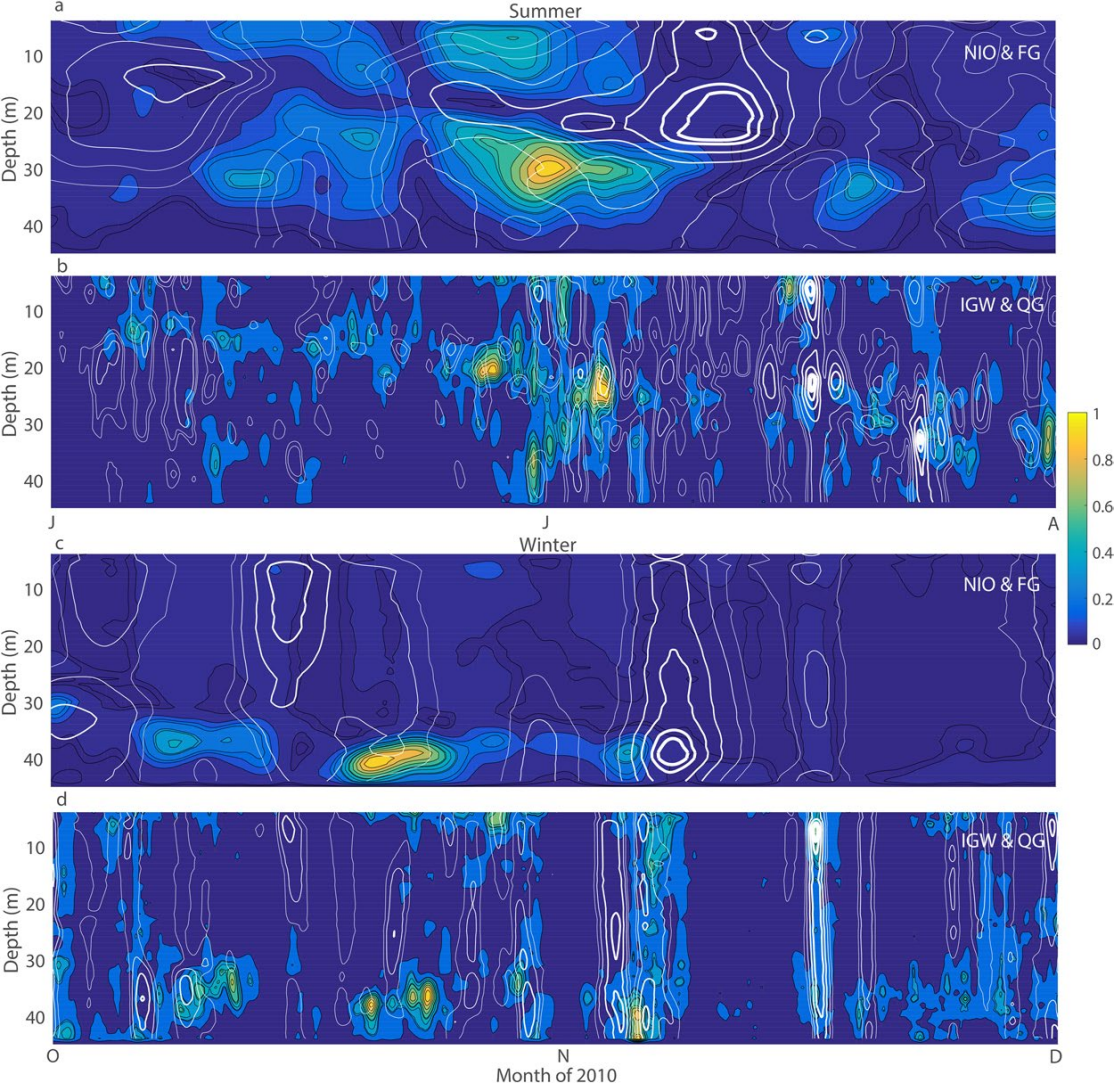
The subinertial current power (white contour lines) overlaid on IIW current power (color-filled contours) for comparison of NIO & FG and IGW & QG current regimes at C12. (**a**) NIO (color-filled) and FG (white) current power during summer and (**c**) winter; (**b**) IGW (color-filled) and QG (white) current power during summer and (**d**) winter. The power is normalized by the maximum value for all variables for simplicity of comparison. The white contours are thicker with greater power magnitude, and the values shown correspond to the normalized power of 0.06, 0.1, 0.2, 0.4, 0.6, 0.8, and 0.9.

Figure 4 illustrates a comparison of the above FG and QG subinertial flow regime estimations with the modulation time scales of the NIO and IGW currents measured in winter (top two panels) and summer (bottom two panels). NIO power is observed to evolve on prominently longer time scales of ~20 days in summer (filled contours in Fig. 4a), whereas in winter the time scales are ~10 days (Fig. 4c). The white FG power contours overlaid on the NIO power contour lines appear to coalign, and NIO power appears to be somewhat larger during weaker FG power in a coherent manner. These observed NIO modulation time scales were confirmed by spectral analyses and are fully consistent with predicted FG time scales in both seasons. Ultimately, the colored contours in Fig. 4b and d show that the IGW power in summer and winter is modulated on approximately similar time scales of ~3–4 days. Although visually less striking but confirmed by spectral estimates, some consistency is apparent between the IGW power contours and white QG power-contour lines, implying that the calculated QG regime time scales are in agreement with the measured IGW time scales in both seasons as well. Therefore, according to Fig. 4, the modulation time scales of the NIO and IGW on WFS are consistent with theoretically predicted (3–5) time scales for subinertial flow in FG and QG regimes.

**Figure 5:**
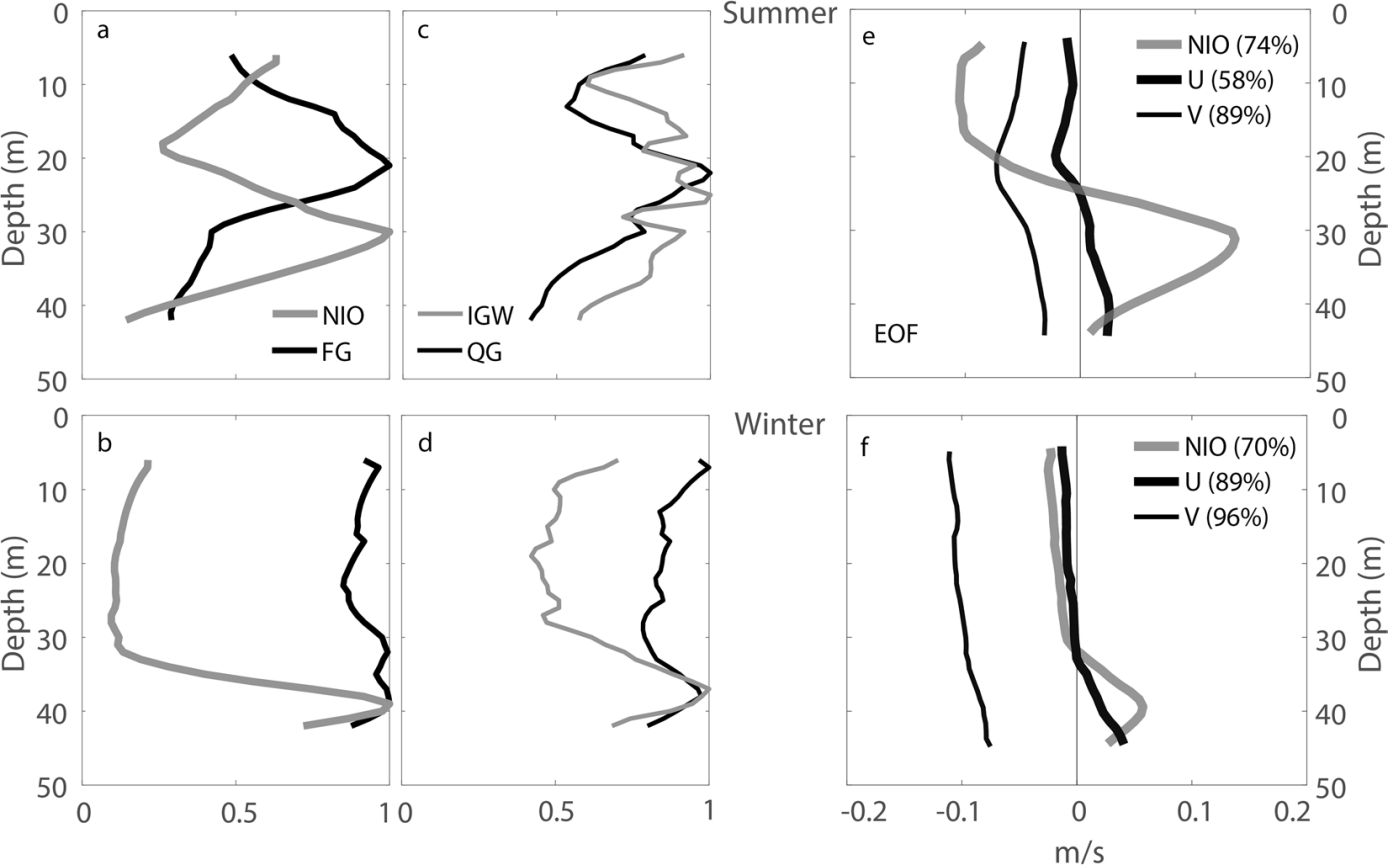
Comparison of the subinertial and IIW time-averaged vertical current structures at C12. (**a**) NIO (grey) and FG (black) average current power during summer and (**b**) winter; (**c**) IGW (grey) and QG (black) average current power during summer and (**d**) winter. The abscissa values are power normalized by the maximum value for all variables. (**e**) First-mode time-domain empirical orthogonal function (EOF) for NIO (grey), subinertial cross-isobath (thick black) and along-isobath (thin black) currents in summer and (**f**) winter. The abscissa values are the standard deviations of the currents at each depth. The variance described by the first-mode EOF is as indicated (%).

Further agreement between the subinertial and IIW currents can be provided by comparison of summer and winter average vertical distributions of the current fluctuations (Fig. 5). The average NIO power (Fig. 5a and b) is largest at ~30 m depth in summer and ~40 m in winter, the levels where the average FG current power appears to be weaker. On the contrary, the average IGW and QG power maxima occupy approximately the same depth, being ~23 m in summer and ~38 m in winter (Fig. 5c and d). Notably, discussed maximum average power depths seasonally decrease from summer to winter for all subinertial and IIW flows. The analysis of the first-mode time-domain empirical orthogonal functions (EOF, Fig. 5e and f) provides more evidence of the seasonality and further associates NIO with the subinertial flow. Both summer and winter EOF structures show 180° phase reversals between the top and bottom NIO fluctuations (grey curves in Fig. 5e and f), a phenomenon that has been routinely observed near other coasts^19^. The average zero crossing in the NIO fluctuations deepens from ~20 m in summer to ~30 m in winter and, remarkably, coincides with the average zero crossings in the subinertial cross-shelf flow fluctuations (thick black curves in Fig. 5e and f).

Overall, the behavior observed in Fig. 5 can be related to seasonal changes in the average stratification, when the average pycnocline position on the shelf deepens in winter because of the thicker wind-driven mixed layer occupying most of the top water column. Indeed, in agreement with previous analysis^20^, the winter along-isobath flow is near-barotropic, except near the bottom (thin black curve in Fig. 5f), whereas the summer along-isobath flow fluctuations present first-baroclinic-mode characteristics (thin black curve in Fig. 5e). According to Fig. 5, therefore, the subinertial and IIW flows on the WFS show concurrent seasonal variations in their vertical structures, which happen in unison and reflect the seasonal changes in the shelf stratification.

## Discussion

The dimensionless analysis and *in situ* measurements on the WFS presented here expose an association between the NIO and FG current regimes corresponding to small *Bu* numbers, and an association between the IGW and QG current regimes corresponding to finite *Bu* numbers. More telling, the NIO and IGW currents on the WFS appear to be modulated on the same time scales as FG and QG subinertial flows, respectively (Fig. 4). In addition, the vertical structures of the subinertial and IIW fluctuations have matching characteristics related to the seasonal changes in stratification (Fig. 5). A thorough analysis is required for full understanding of the dynamic mechanisms^6–9^ that could link the IIW to subinertial flow on the WFS, but some preliminary arguments can be made here.

The FG current power on the WFS appears to be consistent with the low-frequency density changes at corresponding depth levels^13^ - compare the respective months in Figs 2d and 4a. The NIO power could therefore be modulated by the FG evolution of the density fronts by means of frontal-geostrophic adjustment. Such frontal evolution in the region is affected by the winds and processes of upwelling-downwelling, as well as by the influence of the Loop Current and associated eddies^21^. Ultimately, the QG current power is directly coherent with the wind-stress power^13^ - compare the respective months in Figs 2a and 4b,d. The QG flow is therefore probably associated with the synoptic wind-driven currents on the WFS, and these could primarily modulate the IGW in processes of quasi-geostrophic adjustment.

A final remark is as follows. The most widely used oceanic model for surface NIO is a “slab” layer response to the wind forcing over a stationary layer^10^. The model has been shown to have limitations in describing amplitudes and phases of the NIO, particularly below the surface. The model itself could be a source of limitations, because, relying mainly on the wind-stress forcing, it does not account for the adjustment of the full subinertial flow present in the stratified water column. On the other hand, the wind-driven NIO currents near the surface were shown to be modeled with more agreement when the so-called damping term parameter was set to ~10 days. Such time scales are certainly expected to be consistent with the frontal-geostrophic adjustment time scales.

## Methods

The WFS currents have been observed by the long-term bottom-mounted moorings starting in 1998 by the efforts of Ocean Circulation Group at the College of Marine Science of the University of South Florida (http://ocgweb.marine.usf.edu/wfs_current.html). These data have been described in the past^22^. The present study considered records at C10 (20 m), C14 (20 m), and C13 (50 m), but focused on the array of ADCP current-meter records at C12 (50 m) made in 2010, where a more appropriate water stratification record was obtained (Fig. 1). The current measurements at all stations excluded the top and bottom ~10% of the depth as being contaminated by side lobe interactions near boundaries.

The near-continuous 3-hourly *in situ* water-column density is available at C12 (1, 10, 20, and 30 m depth) and calculated from temperature and salinity data by means of the 2010 International Thermodynamic Equation of Seawater^23^ with the GSWv3.5 software package provided by the consortium.

The offshore atmospheric data are available at the National Oceanic and Atmospheric Administration’s National Data Buoy Center (NOAA NDBC) station 42039 southeast of Pensacola, Florida (http://www.ndbc.noaa.gov/station_page.php?station=42039). The wind-stress components are calculated from hourly wind-speed and wind-direction time series^24^.

After initial quality control and barotropic tide filtering^25^, all current and wind-stress time series were low-pass or band-pass filtered by means of a cosine-Lanczos filter^26^ . The central WFS is located near the critical latitude 30°N, where the tidal diurnal periods (K1 23.93 h, O1 25.82 h) are close to the inertial period (~26–27 h at the central WFS latitudes around 27.5°N). In particular, the inertial period at C12 is 25.96 h, corresponding to the inertial frequency 0.92 cycles/day. The low-frequency (subinertial) band is therefore defined as all subinertial fluctuations at periods longer than 40 h (the filter passes 50% power at 40 h and 5% power at 30 h). In accordance with the dimensionless analysis and *in situ Fr* estimations presented above, the subinertial band is further divided into a low-pass filtered FG band (periods longer than 8 days in winter and 17 days in summer), and the QG band being the remaining low-frequency band-pass filtered part (40 h to 8 or 17 days in winter or summer, respectively). Finally, analogical filters include the IIW band-pass filter constructed to isolate the currents at the inertial and superinertial frequencies (5–27 h), the NIO band-pass filter constructed to isolate the inertial period (24–27 h), and last, the IGW band-pass filter constructed to isolate the superinertial fluctuations (5–18 h).

The power spectral density is estimated according to a multitaper spectral method^27^ with 4 Slepian tapers. The spectral significance is tested at the 95% confidence level against a red-noise background. Confidence levels are not shown in the figures for clarity, but the peaks discussed in the text are significant at 95% confidence level. Finally, the power of the current signal is calculated by continuous wavelet transform with Morlet wavelets with account for bias^26,28^. The power in a given frequency band (IIW, NIO, IGW, Subinertial, FG, and QG) is calculated by integrating the wavelet scalogram over that frequency band.
